# Skating on thin ice: stimulant use and sub‐optimal adherence to HIV pre‐exposure prophylaxis

**DOI:** 10.1002/jia2.25103

**Published:** 2018-03-25

**Authors:** J Carlo Hojilla, David Vlahov, David V Glidden, K Rivet Amico, Megha Mehrotra, Robert Hance, Robert M Grant, Adam W Carrico

**Affiliations:** ^1^ University of California San Francisco San Francisco CA USA; ^2^ Yale University West Haven CT USA; ^3^ University of Michigan Ann Arbor MI USA; ^4^ J. David Gladstone Institutes San Francisco CA USA; ^5^ University of Miami Miami FL USA

**Keywords:** pre‐exposure prophylaxis, adherence, drug use, stimulant use, binge drinking, men who have sex with men, transgender persons

## Abstract

**Introduction:**

Stimulant and heavy alcohol use are prevalent and associated with elevated risk for HIV seroconversion among men who have sex with men (MSM) and transgender women. In addition, each can pose difficulties for antiretroviral adherence among people living with HIV. Scant research has examined the associations of stimulant and heavy alcohol use with adherence to daily oral pre‐exposure prophylaxis (PrEP) among MSM and transgender women. To address this gap in the literature, we evaluated the hypothesis that stimulant use and binge drinking are prospectively associated with sub‐optimal PrEP adherence.

**Methods:**

We analysed data from participants in a nested case‐cohort in the iPrEx open label extension. Stimulant use (i.e. powder cocaine, crack‐cocaine, cocaine paste, methamphetamine, cathinone) and binge drinking (i.e. ≥5 drinks in a single day) in the last 30 days were assessed. Baseline urine was tested for stimulants using immunoassays to reduce misclassification. Sub‐optimal adherence was defined as tenofovir drug concentrations in dried blood spots less than 700 fmol per punch, indicative of less than four doses per week. We tested the prospective association of stimulant use and binge drinking with sub‐optimal adherence at the 4‐week follow‐up visit.

**Results and Discussion:**

Data from 330 participants were analysed. The majority of the participants were MSM (89%) with a median age at baseline of 29 years (interquartile range 24 to 39). Approximately 16% (52/330) used stimulants and 22% (72/330) reported binge drinking in the last 30 days. Stimulant users had fivefold greater odds of sub‐optimal PrEP adherence compared to non‐users in adjusted analysis (adjusted odds ratio [aOR] 5.04; [95% CI 1.35 to 18.78]). Self‐reported binge drinking was not significantly associated with sub‐optimal adherence after adjusting for stimulant use and baseline confounders (aOR 1.16 [0.49 to 2.73]). Depressive symptoms, being transgender, and number of sex partners were also not significantly associated with sub‐optimal PrEP adherence (*p *>* *0.05).

**Conclusions:**

Stimulant use is a risk factor for sub‐optimal PrEP adherence in the month following PrEP initiation. Comprehensive prevention approaches that reduce stimulant use may optimize PrEP adherence. Creating adherence plans that specifically address PrEP dosing in the context of ongoing stimulant use should also be considered.

## 1 Introduction

Men who have sex with men (MSM) are 24 times more likely to become infected with HIV compared to the general population, while transgender women are nearly 50 times more likely to become infected 1. Daily oral HIV pre‐exposure prophylaxis (PrEP) using emtricitabine‐tenofovir disoproxyl fumarate (FTC‐TDF) has demonstrated efficacy in preventing HIV infection 2, 3, 4, 5, 6 and is a promising strategy for these key populations. In the iPrEx trial 2, MSM and transgender women taking PrEP had an overall 44% reduction in HIV acquisition. More recent data from the PROUD study 6 reported a relative reduction of 86% among individuals taking daily oral PrEP, suggesting that this strategy is highly effective in preventing HIV in high priority populations.

However, the unprecedented clinical and public health benefits of PrEP require sustained, prevention effective levels of adherence during periods of exposure to HIV 3, 7. In the iPrEx Open Label Extension (OLE), drug concentrations in dried blood spots (DBS) corresponding to four or more doses of FTC‐TDF per week (i.e. ≥700 fmol per punch) were associated with no new HIV infections 3. But despite high interest and uptake of PrEP among MSM and transgender women 8, 9, a considerable proportion of individuals either discontinue or struggle to adhere to the regimen shortly after initiation 3, 10. In several studies, all acute HIV seroconversions occurred exclusively during periods of inadequate PrEP adherence or when PrEP was discontinued 3, 4, 7.

Existing studies have not clearly elucidated the reasons for sub‐optimal PrEP adherence among MSM and transgender women 11, but alcohol and stimulant use may be important risk factors. Stimulant and heavy alcohol use are well‐established correlates of HIV acquisition and poor treatment outcomes among HIV‐positive individuals 12, 13. There is also increasing recognition that “chemsex,” or the use of a combination of sex enhancing drugs like stimulants, has become increasingly common among MSM residing in industrialized nations 14, 15. In a large observational study of MSM, stimulant use was independently associated with a threefold increase in risk for HIV seroconversion and an eightfold increase when combined with other chemsex drugs such as poppers 16. In the United States, estimates of stimulant use range between 6% and 17% among MSM 17 and between 21% and 26% among transgender women 18, 19, 20. Reports also suggest high rates of alcohol use in these individuals (approximately 56% in MSM and 44% in transgender women) 17, 19. However, the associations of stimulant use and heavy alcohol use with sub‐optimal PrEP adherence is not well understood. To address this gap in knowledge, this study evaluated the hypothesis that stimulant use and binge drinking at baseline are prospectively associated with early sub‐optimal PrEP adherence in an observational cohort of MSM and transgender women.

## 2 Methods

### 2.1 Study participants

We analysed data from participants in a nested case‐cohort in iPrEx OLE. Individuals in the case‐cohort had drug concentrations measured at each time point after PrEP dispensation. The parent study and the design of the case‐cohort are described in detail elsewhere 3, 21. Briefly, participants were enrolled at 11 sites across six countries (Brazil, Ecuador, Peru, South Africa, Thailand and the United States) between June 2011 and June 2012, and followed for up to 72 weeks. Visits were scheduled 4, 8 and 12 weeks after enrolment, and quarterly thereafter. Participants were male sex at birth, reported having anal sex with men, and were at least 18 years of age. All participants provided informed consent as part of the parent study. The iPrEx OLE study protocol was approved by institutional review boards at each site and by relevant national regulatory agencies. This study was approved by the Committee on Human Research at the University of California, San Francisco.

### 2.2 Measures

#### 2.2.1 Stimulant use and binge drinking

Participants reported any stimulant use and binge drinking in the last 30 days using computer‐assisted self‐interview (CASI). Stimulants included powder cocaine, crack‐cocaine, cocaine paste, methamphetamine, and the amphetamine analogue, cathinone. Immunochemical screening of banked urine collected at baseline was used to validate self‐reported stimulant use. Participants who denied any recent stimulant use but had a positive urine immunoassay were coded as having used stimulants. Binge drinking was defined as consuming ≥5 alcoholic drinks in a single day by self‐report.

#### 2.2.2 Depressive symptoms

Depressive symptoms at baseline were measured using the Centre for Epidemiologic Studies Depression (CES‐D) scale, a validated 20‐item measure that assesses for the frequency of depressive symptoms in the past week 22. Standard clinical cut‐offs were used to categorize CES‐D scores: none‐mild depression (0 to 15); mild‐moderate depression (16 to 26); and severe depression (≥27).

#### 2.2.3 Sexual risk behaviours

Condomless sex and the number of sex partners in the last three months were assessed at baseline using an interviewer‐administered questionnaire.

#### 2.2.4 Sub‐optimal PrEP adherence

Participants in the case‐cohort had PrEP adherence estimated by tenofovir diphosphate (TFV‐DP) concentrations in DBS, as described previously 3. TFV‐DP concentrations <700 fmol per punch corresponds to an average of less than four doses per week. This dosing pattern provides sub‐optimal protection against HIV 3.

### 2.3 Analysis

We tested the direct association of baseline stimulant use and binge drinking with sub‐optimal PrEP adherence at the 4‐week follow‐up visit using logistic models. Probability weights were used to account for the case‐cohort design. As a sensitivity analysis, we repeated our analysis using a robust variance estimator to account for clustering within site. Because there were no observed differences in effect size estimates, we report analyses that do not adjust for clustering given the relatively modest number of sites in this project. We evaluated PrEP adherence at the 4‐week follow‐up visit based on previous analysis that demonstrated that poor adherence at this visit was highly predictive of future adherence patterns 23. Relevant baseline confounders were identified using a directed acyclic graph 24. Each predictor was modelled individually then as a predictor set. Covariate selection for the final model was based on changes in statistical precision 24. Any sexually transmitted infection at enrolment was omitted in the final model as this resulted in a substantially larger standard error. Given the high prevalence of depression among MSM and transgender women 25, 26 and the bivariate association between depression and non‐adherence 27, 28, we tested for the possible interaction between our predictors of interest and baseline CES‐D score. We also tested for the possible interaction between stimulant use and binge drinking. However, we found no evidence of effect heterogeneity (*p *>* *0.05) or any qualitatively meaningful changes in effect size estimates, so these terms were not included in the final model. All analyses were conducted using Stata 14 (College Station, TX).

## 3 Results

### 3.1 Sample characteristics

A total of 349 participants were included in the iPrEx OLE case‐cohort. Data from 330 participants who provided consent for long‐term specimen banking and testing were included in this analysis (Table 1). Median age at baseline was 29 years (interquartile range [IQR] 24 to 39) and median number of sex partners in the last three months was two (IQR 1 to 5). Most identified as MSM (89%), Latino or Hispanic (57%), and had completed at least secondary education (78%). Approximately 16% (52/330) used stimulants and 22% (72/330) reported binge drinking in the last month. Nearly half (49%) of all participants reported engaging in any condomless sex in the previous three months at baseline.

**Table 1:** Baseline demographics and participant characteristics (N = 330)

	Median	(IQR)
Age	29	(24 to 39)
Total number of partners in last 3 months	2	(1 to 5)
	*n*	(%)
Study region		
Andes^a^	165	(50)
Brazil	68	(21)
South Africa	19	(6)
Thailand	18	(5)
United States	60	(18)
Men who have sex with men	295	(89)
Transgender women	35	(11)
Latino or Hispanic	187	(57)
Education		
Less than secondary	71	(22)
Completed secondary	106	(32)
Post‐secondary	152	(46)
Baseline CES‐D^b^ score		
<16	237	(72)
16 to 26	63	(19)
≥27	29	(9)
Stimulant use in the last 30 days^c^	52	(16)
Binge in the last 30 days^d^	72	(22)
Condomless anal sex in the last 3 months	161	(49)

IQR, interquartile range.

a Includes sites in Ecuador and Peru.

b Centre for Epidemiologic Studies Depression scale.

c Stimulant use based on self‐report or positive urine immunoassay. Includes powder cocaine, crack‐cocaine, cocaine paste, methamphetamine and cathinone (amphetamine analogue).

d ≥5 alcoholic drinks in a single day.

### 3.2 PrEP adherence

TFV‐DP concentrations in DBS at the 4‐week follow‐up were available in 293 participants. Of those with available TFV‐DP data, approximately 47% (137/293) had drug concentrations indicative of less than four doses in the prior week. Approximately 55% of stimulant users (24/44) and binge drinkers (34/62) were sub‐optimally adherent at the 4‐week follow‐up visit.

In adjusted analysis, stimulant use was significantly associated with sub‐optimal PrEP adherence (Table 2). Stimulant users were five times more likely to have TVF‐DP concentrations <700 fmol per punch at the 4‐week follow‐up compared to non‐users (adjusted odds ratio [aOR] 5.04; [95% CI: 1.35 to 18.78]). Binge drinking was not significantly associated with sub‐optimal adherence (aOR 1.16 [0.49 to 2.73]). Total number of partners in the last three months and depressive symptoms were also not significantly associated with sub‐optimal PrEP adherence. We found no statistically significant linear trend for total number of partners (*p *=* *0.87) and CES‐D score (*p *=* *0.79) in the multivariable model. When we limited our analysis to only MSM, we observed a slightly larger association of stimulant use on sub‐optimal adherence (aOR 8.82 [2.05 to 37.90]). The association of binge drinking remained non‐significant (aOR 1.31 [0.52 to 3.34]).

**Table 2:** Baseline correlates of sub‐optimal PrEP adherence at the 4‐week follow‐up visit (n = 293)

	Adjusted OR	(95% CI)	*p*‐value
Stimulant use in the last 30 days^a^	5.04	(1.35 to 18.78)	0.02
Binge drinking in the last 30 days^b^	1.16	(0.49 to 2.73)	0.74
Transgender	1.24	(0.20 to 7.58)	0.82
Total number of partners in the last 3 months			
0 to 1 partners	Ref		
2 to 3 partners	0.78	(0.25 to 2.40)	0.67
≥4 partners	0.91	(0.28 to 2.99)	0.87
CES‐D score^c^			
<16	Ref		
16 to 26	0.43	(0.15 to 1.18)	0.10
≥27	1.18	(0.34 to 4.11)	0.79

Multivariable model also controlled for study region, age, education, and Latino or Hispanic ethnicity. Sub‐optimal adherence is defined as tenofovir diphosphate (TFV‐DP) concentrations <700 fmol per punch based on dried blood spots (DBS), corresponding to <4 doses per week. All predictors were assessed at the baseline visit. We found no statistically significant linear trend for total number of partners (*p* = 0.87) and CES‐D score (*p* = 0.79) in the multivariable model.

a Stimulant use based on self‐report or positive urine immunoassay. Includes powder cocaine, crack‐cocaine, cocaine paste, methamphetamine and cathinone (amphetamine analogue).

b Binge drinking is ≥5 alcoholic drinks in a single day.

c Centre for Epidemiologic Studies Depression (CES‐D) scale.

## 4 Discussion

This study is among the first to document that stimulant use is associated with a fivefold greater odds of sub‐optimal PrEP adherence, measured by TFV‐DP drug concentrations below the established threshold to maximize protection against HIV. The association between stimulant use and poor adherence to antiretroviral therapy is well‐described in the HIV treatment literature 12, 29, 30, and findings from this study highlight that stimulant use may also undermine the clinical and public health benefits of PrEP.

PrEP implementation has slowly gained traction in recent years and data from real‐world clinical experiences have shown encouraging results. Recent studies have reported high demand for PrEP in various clinical settings 31, 32 and PrEP implementation outcomes have seen high overall rates of retention over time 33, 34, 35. In a study examining retention in care outcomes in a community‐based sexual health clinic in San Francisco, Hojilla et al. 35 found that stimulant use and unhealthy drinking behaviours were not significantly associated with PrEP discontinuation. However, an important limitation of the study was its limited ability to accurately measure PrEP adherence and recent drug and alcohol use. Because PrEP implementation is in its early stages 36, 37, 38, studies from clinical settings likely involve a large proportion of highly motivated early adopters that are not representative of the broader population of MSM and transgender women. Thus, important questions around potential barriers to optimal PrEP adherence in this key population remain unanswered. Although the field of PrEP research has advanced considerably since iPrEx OLE, data from this study remain particularly salient in the context of ongoing discussions around scale‐up and developing strategies to optimize PrEP delivery with high priority populations.

Our findings support the need to provide PrEP as part of a comprehensive package that includes an assessment of potential barriers and interventions to enhance PrEP adherence. Buchbinder and colleagues' 39 secondary analysis of data from the randomized phase of iPrEX estimated that the lowest number needed to treat (NNT) occurred specifically among MSM and transgender women who used stimulants (NNT = 12). Thus, engaging stimulant users in the PrEP continuum is a high priority. Our work suggests that for PrEP to achieve its maximum clinical and public health impact, PrEP delivery models should incorporate efforts to optimize adherence in stimulant‐using populations.

Focused work is needed to identify evidence‐based interventions that can mitigate sub‐optimal PrEP adherence in stimulant‐using MSM and transgender women, particularly in the first month of starting the regimen. Expanded efforts with stimulant users could leverage intensive case management and follow‐up to support PrEP adherence. Integration of evidence‐based interventions that directly reduce stimulant use could also achieve meaningful improvements in PrEP adherence. For example, motivational interviewing and cognitive‐behavioural interventions have demonstrated efficacy in reducing substance use and sexual risk taking in MSM 40, 41. Clinical research to develop and test interventions to optimize the prevention effectiveness of PrEP in stimulant users could achieve more meaningful reductions in HIV incidence.

This study has some limitations. Self‐reported responses may have been prone to recall and social desirability biases, particularly for sensitive topics like alcohol and other substance use. We attempted to mitigate misclassification using urine biomarkers of stimulant use to validate participant responses, but this may have been incomplete based upon the limited window of detection for recent stimulant use. We were also unable to differentiate between frequent and episodic stimulant and alcohol use, which may influence PrEP adherence in a dose–response manner. Binge drinking was operationalized as five or more alcoholic drinks in a single day rather than in one 2‐hour sitting 42, which may have overestimated the number of individuals classified as binge drinkers in our study. As sexual risk can fluctuate over time 43, it is possible that some individuals only took PrEP during periods of possible exposure (e.g. on‐demand dosing). Alternative dosing strategies might account for sub‐optimal adherence in some participants. However, all individuals were counselled on the importance of daily PrEP use given the limited data available at the time on intermittent or on‐demand PrEP use. Lastly, we acknowledge that transgender women are a distinct group of individuals that do not necessarily share the same psychosocial contexts as MSM. Our study was not sufficiently powered to detect differences between these groups. Future studies will need to examine transgender persons separately to clearly elucidate factors associated with PrEP adherence in this population.

## 5 Conclusions

In this study, we utilized biomarkers of stimulant use to reduce misclassification and examined a validated biomarker of PrEP adherence as the outcome. Our findings demonstrate that stimulant use is a risk factor for sub‐optimal PrEP adherence in the month following initiation. Identification of stimulant use as a risk factor for the impaired prevention effectiveness of PrEP is a crucial first step to inform the development of comprehensive approaches to optimize its clinical and public health benefits in high priority populations.

## Competing interests

DVG has received fees from Gilead Sciences. The other authors have no competing interests to declare.

## Authors' contributions

A.C., K.R.M. and J.C.H contributed to study concept and design. J.C.H., A.C., D.V., R.H., M.M., D.V.G. and R.M.G contributed to data retrieval, analysis, or interpretation. J.C.H and A.C. contributed to manuscript preparation. All authors provided critical review and edits. The final manuscript was approved by all authors.

## References

[1] UNAIDS . Prevention gap report. Geneva, Switzerland; 2016. Available from: http://www.unaids.org/sites/default/files/media_asset/2016-prevention-gap-report_en.pdf [cited 2017 Aug 12]

[2] Grant RM , Lama JR , Anderson PL , McMahan V , Liu AY , Vargas L , et al. Preexposure chemoprophylaxis for HIV prevention in men who have sex with men. N Engl J Med. 2010;363(27):2587–99. 21091279 10.1056/NEJMoa1011205 PMC3079639

[3] Grant RM , Anderson PL , McMahan V , Liu A , Amico KR , Mehrotra M , et al. Uptake of pre‐exposure prophylaxis, sexual practices, and HIV incidence in men and transgender women who have sex with men: a cohort study. Lancet Infect Dis. 2014;14(9):820–9. 25065857 10.1016/S1473-3099(14)70847-3 PMC6107918

[4] Liu AY , Cohen SE , Vittinghoff E , Anderson PL , Doblecki-Lewis S , Bacon O , et al. Preexposure prophylaxis for HIV infection integrated with municipal‐ and community‐based sexualhealth services. JAMA Intern Med. 2016;176(1):11–75. 26571482 10.1001/jamainternmed.2015.4683 PMC5042323

[5] Choopanya K , Martin M , Suntharasamai P , Sangkum U , Mock PA , Leethochawalit M , et al. Antiretroviral prophylaxis for HIV infection in injecting drug users in Bangkok, Thailand (the Bangkok Tenofovir Study): a randomised, double‐blind, placebo‐controlled phase 3 trial. Lancet. 2013;381(9883):2083–90. 23769234 10.1016/S0140-6736(13)61127-7

[6] McCormack S , Dunn DT , Desai M , Dolling DI , Gafos M , Gilson R , et al. Pre‐exposure prophylaxis to prevent the acquisition of HIV‐1 infection (PROUD): effectiveness results from the pilot phase of a pragmatic open‐label randomised trial. Lancet. 2016;387(10013):53–60. 26364263 10.1016/S0140-6736(15)00056-2 PMC4700047

[7] Molina J-M , Charreau I , Spire B , Cotte L , Chas J , Capitant C , et al. Efficacy, safety, and effect on sexual behaviour of on‐demand pre‐exposure prophylaxis for HIV in men who have sex with men: an observational cohort study. Lancet HIV. 2017;4(9):e402–10. 28747274 10.1016/S2352-3018(17)30089-9

[8] Cohen SE , Vittinghoff E , Bacon O , Doblecki-Lewis S , Postle BS , Feaster DJ , et al. High interest in preexposure prophylaxis among men who have sex with men at risk for HIV infection: baseline data from the US PrEP demonstration project. J Acquir Immune Defic Syndr. 2015;68(4):439–48. 25501614 10.1097/QAI.0000000000000479 PMC4334721

[9] Sevelius JM , Keatley J , Calma N , Arnold E . “I am not a man”: trans‐specific barriers and facilitators to PrEP acceptability among transgender women. Glob Public Health. 2016;11(7–8):1060–75. 26963756 10.1080/17441692.2016.1154085 PMC10204128

[10] Deutsch MB , Glidden DV , Sevelius J , Keatley J , McMahan V , Guanira J , et al. HIV pre‐exposure prophylaxis in transgender women: a subgroup analysis of the iPrEx trial. Lancet HIV. 2015;2(12):e512–9. 26614965 10.1016/S2352-3018(15)00206-4 PMC5111857

[11] Blashill AJ , Ehlinger PP , Mayer KH , Safren SA . Optimizing adherence to preexposure and postexposure prophylaxis: the need for an integrated biobehavioral approach. Clin Infect Dis. 2015;60(suppl 3):S187–90. 25972502 10.1093/cid/civ111 PMC4551106

[12] Carrico AW , Riley ED , Johnson MO , Charlebois ED , Neilands TB , Remien RH , et al. Psychiatric risk factors for HIV disease progression: the role of inconsistent patterns of antiretroviral therapy utilization. J Acquir Immune Defic Syndr. 2011;56(2):146–50. 21116186 10.1097/QAI.0b013e318201df63 PMC3494991

[13] Plankey MW , Ostrow DG , Stall R , Cox C , Li X , Peck JA , et al. The relationship between methamphetamine and popper use and risk of HIV seroconversion in the multicenter AIDS cohort study. J Int AIDS Soc. 2007;45(1):85–92. 10.1097/QAI.0b013e3180417c99 PMC3486782 17325605

[14] Kirby T , Thornber-Dunwell M . High‐risk drug practices tighten grip on London gay scene. Lancet. 2013;381(9861):101–2. 23320280 10.1016/s0140-6736(13)60032-x

[15] Sewell J , Miltz A , Lampe FC , Cambiano V , Speakman A , Phillips AN , et al. Poly drug use, chemsex drug use, and associations with sexual risk behaviour in HIV‐negative men who have sex with men attending sexual health clinics. Int J Drug Policy. 2017;43:33–43. 28189979 10.1016/j.drugpo.2017.01.001

[16] Ostrow DG , Plankey MW , Cox C , Li X , Shoptaw S , Jacobson LP , et al. Specific sex drug combinations contribute to the majority of recent HIV seroconversions among MSM in the MACS. J Acquir Immune Defic Syndr. 2009;51(3):349–55. 19387357 10.1097/QAI.0b013e3181a24b20 PMC3074969

[17] Centers for Disease Control and Prevention . HIV infection risk, preventing, and testing behaviors among men who have sex with men - National HIV behavioral surveillance, 20 U.S. cities, 2014. [Internet]. Vol. 15. 2016 Available from: https://www.cdc.gov/hiv/pdf/library/reports/surveillance/cdc-hiv-hssr-nhbs-msm-2014.pdf [cited 2018 Feb 18]

[18] Flentje A , Heck NC , Sorensen JL . Characteristics of transgender individuals entering substance abuse treatment. Addict Behav. 2014;39(5):969–75. 24561017 10.1016/j.addbeh.2014.01.011 PMC4130569

[19] Herbst JH , Jacobs ED , Finlayson TJ , McKleroy VS , Neumann MS , Crepaz N , et al. Estimating HIV prevalence and risk behaviors of transgender persons in the United States: a systematic review. AIDS Behav. 2008;12(1):1–17. 17694429 10.1007/s10461-007-9299-3

[20] Reback CJ , Fletcher JB . HIV prevalence, substance use, and sexual risk behaviors among transgender women recruited through outreach. AIDS Behav. 2014;18(7):1359–67. 24287786 10.1007/s10461-013-0657-z PMC4209944

[21] Prentice RL . A case‐cohort design for epidemiologic cohort studies and disease prevention trials. Biometrika. 1986;73(1):1–11.

[22] Radloff LS . The CES‐D Scale. Appl Psychol Meas. 1977;1(3):385–401.

[23] Glidden DV , Buchbinder SP , Anderson PL , McMahan V , Amico KR , Liu AY , et al. PrEP engagement for HIV prevention: Results from the iPrEx Open Label Extension. In: Conference on Retroviruses and Opportunistic Infections; 2015 Feb 23-26. Boston, USA. Available from: http://www.croiconference.org/sites/default/files/posters-2015/970.pdf

[24] Weng H-Y , Hsueh Y-H , Messam LLM , Hertz-Picciotto I . Methods of covariate selection: directed acyclic graphs and the change‐in‐estimate procedure. Am J Epidemiol. 2009;169(10):1182–90. 19363102 10.1093/aje/kwp035

[25] Meyer IH , Dietrich J , Schwartz S . Lifetime prevalence of mental disorders and suicide attempts in diverse lesbian, gay, and bisexual populations. Am J Public Health. 2008;98(6):1004–6. 17901444 10.2105/AJPH.2006.096826 PMC2377299

[26] Defechereux PA , Mehrotra M , Liu AY , McMahan VM , Glidden DV , Mayer KH , et al. Depression and oral FTC/TDF pre‐exposure prophylaxis (PrEP) among men and transgender women who have sex with men (MSM/TGW). AIDS Behav. 2016;20(7):1478–88. 26078115 10.1007/s10461-015-1082-2 PMC4903104

[27] Gonzalez JS , Batchelder AW , Psaros C , Safren SA . Depression and HIV/AIDS treatment nonadherence: a review and meta‐analysis. J Acquir Immune Defic Synd. 2011;58(2):181–7. 10.1097/QAI.0b013e31822d490a PMC3858003 21857529

[28] Mehrotra ML , Glidden DV , McMahan V , Amico KR , Hosek S , Defechereux P , et al. The effect of depressive symptoms on adherence to daily oral PrEP in men who have sex with men and transgender women: a marginal structural model analysis of the iPrEx OLE study. AIDS Behav. 2016;20(7):1527–34. 27125241 10.1007/s10461-016-1415-9 PMC4912836

[29] Hinkin CH , Barclay TR , Castellon SA , Levine AJ , Durvasula RS , Marion SD , et al. Drug use and medication adherence among HIV‐1 infected individuals. AIDS Behav. 2007;11(2):185–94. 16897351 10.1007/s10461-006-9152-0 PMC2867605

[30] Marquez C , Mitchell SJ , Hare CB , John M , Klausner JD . Methamphetamine use, sexual activity, patient‐provider communication, and medication adherence among HIV‐infected patients in care, San Francisco 2004‐2006. AIDS Care. 2009;21(5):575–82. 19444665 10.1080/09540120802385579

[31] Volk JE , Marcus JL , Phengrasamy T , Blechinger D , Nguyen DP , Follansbee S , et al. No new HIV infections with increasing use of HIV preexposure prophylaxis in a clinical practice setting. Clin Infect Dis. 2015;61(10):1601–3. 26334052 10.1093/cid/civ778 PMC4809999

[32] Liu A , Cohen S , Follansbee S , Cohan D , Weber S , Sachdev D , et al. Early experiences implementing pre‐exposure prophylaxis (PrEP) for HIV prevention in San Francisco. PLoS Med. 2014;11(3):e1001613. 24595035 10.1371/journal.pmed.1001613 PMC3942317

[33] Marcus JL , Hurley LB , Hare CB , Nguyen DP , Phengrasamy T , Silverberg MJ , et al. Preexposure prophylaxis for HIV prevention in a large integrated health care system: adherence, renal safety, and discontinuation. J Acquir Immune Defic Synd. 2016;73(5):540–6. 10.1097/QAI.0000000000001129 PMC5424697 27851714

[34] Chan PA , Mena L , Patel R , Oldenburg CE , Beauchamps L , Perez-Brumer AG , et al. Retention in care outcomes for HIV pre‐exposure prophylaxis implementation programmes among men who have sex with men in three US cities. J Int AIDS Soc. 2016;19(1):20903. 27302837 10.7448/IAS.19.1.20903 PMC4908080

[35] Hojilla JC , Vlahov D , Crouch PC , Dawson-Rose C , Freeborn K , Carrico A . HIV pre‐exposure prophylaxis (PrEP) uptake and retention among men who have sex with men in a community‐based sexual health clinic. AIDS Behav. 2017;31(3):731. 10.1007/s10461-017-2009-x PMC5879003 29243109

[36] Coleman R , Prins M . Options for affordable pre‐exposure prophylaxis (PrEP) in national HIV prevention programmes in Europe. Euro Surveill. 2017;22(42):2179. 10.2807/1560-7917.ES.2017.22.42.17-00698 PMC5710114 29067904

[37] Mayer KH , Chan PA , R Patel R , Flash CA , Krakower DS . Evolving models and ongoing challenges for HIV preexposure prophylaxis implementation in the United States. J Acquir Immune Defic Synd 2018;77(2):119–27. 10.1097/QAI.0000000000001579 PMC5762416 29084044

[38] Cowan FM , Delany-Moretlwe S , Sanders EJ , Mugo NR , Guedou FA , Alary M , et al. PrEP implementation research in Africa: what is new? J Int AIDS Soc. 2016;19 7 Suppl 6: 21101. 27760680 10.7448/IAS.19.7.21101 PMC5071780

[39] Buchbinder SP , Glidden DV , Liu AY , McMahan V , Guanira JV , Mayer KH , et al. HIV pre‐exposure prophylaxis in men who have sex with men and transgender women: a secondary analysis of a phase 3 randomised controlled efficacy trial. Lancet Infect Dis. 2014;14(6):468–75. 24613084 10.1016/S1473-3099(14)70025-8 PMC4133171

[40] Shoptaw S , Reback CJ . Methamphetamine use and infectious disease‐related behaviors in men who have sex with men: implications for interventions. Addiction. 2007;102(Suppl 1):130–5. 17493062 10.1111/j.1360-0443.2006.01775.x

[41] Carrico AW , Zepf R , Meanley S , Batchelder A , Stall R . Critical review: when the party is over: a systematic review of behavioral interventions for substance‐using men who have sex with men. J Acquir Immune Defic Synd. 2016;73(3):299–306. 10.1097/QAI.0000000000001102 PMC5065362 27258233

[42] National Institute of Alcohol Abuse and Alcoholism . NIAAA council approves definition of binge drinking. 2004 Available from: https://pubs.niaaa.nih.gov/publications/Newsletter/winter2004/Newsletter_Number3.pdf [cited 2018 Feb 18]

[43] Hojilla JC , Koester KA , Cohen SE , Buchbinder S , Ladzekpo D , Matheson T , et al. Sexual behavior, risk compensation, and HIV prevention strategies among participants in the San Francisco PrEP Demonstration Project: a qualitative analysis of counseling notes. AIDS Behav. 2015;20:1–9. 10.1007/s10461-015-1055-5 PMC4592687 25835463

